# Computer-aided resection and endoprosthesis design for the management of malignant bone tumors around the knee: outcomes of 12 cases

**DOI:** 10.1186/1471-2474-14-331

**Published:** 2013-11-22

**Authors:** Huan-wen Ding, Guang-wen Yu, Qiang Tu, Bao Liu, Jian-jian Shen, Hong Wang, Ying-jun Wang

**Affiliations:** 1 School of Materials Science and Engineering, South China University of Technology, Guangzhou, Guangdong 510010, China; 2 Department of Overseas Chinese, General Hospital of Guangzhou Command of PLA, No.111 LiuHua Road, Guangzhou 510010, Guangdong, China

**Keywords:** Computer aided design, Bone tumor, Individual bone restoration, Individualized prosthesis

## Abstract

**Background:**

To report the outcomes of computer-aided resection and endoprosthesis design for the management of malignant bone tumors around the knee.

**Methods:**

Computed tomography (CT) and magnetic resonance imaging (MRI) data were input into computer software to produce three-dimensional (3D) models of the tumor extent. Imaging data was then used to create a template for surgical resection, and development of an individualized combined allogeneic bone/endoprosthesis. Surgical simulations were performed prior to the actual surgery.

**Results:**

This study included 9 males and 3 females with a mean age of 25.3 years (range, 13 to 40 years). There were 9 tumors in the distal femur and 3 in the proximal tibia. There were no surgical complications. In all cases pathologically confirmed clear surgical margins were obtained. Postoperative radiographs showed the range of tumor resection was in accordance with the preoperative design, and the morphological reconstruction of the bone defect was satisfactory with complete bilateral symmetry. The mean follow-up time was 26.5 months. Two patients died of their disease and the remaining are alive and well without evidence of recurrence. All patients are able to ambulate freely without restrictions. At the last follow-up, the average International Society of Limb Salvage score was 25.8 (range, 18 to 27), and was excellent in 8 cases and good in 4 cases.

**Conclusions:**

Computer-aided design and modeling for the surgical management of bone tumors and subsequent limb reconstruction provides accurate tumor removal with the salvage of a maximal amount of unaffected bone and precise endoprosthesis reconstruction.

## Background

Osteosarcoma is the most common primary bone tumor, and approximately 70% of osteosarcomas occur around the knee joint [1,2]. Though in the past bone sarcomas were primarily treated with amputation, advances in surgical techniques and chemotherapy have greatly improved the prognosis, and currently long-term disease free survival rates for patients with osteosarcomas with no metastases at presentation range from 60 to 80% [3]. Limb salvage surgery has replaced amputation as the primary surgical treatment with the goal of surgery to achieve a normal margin of tissue around the pseudo capsule of the tumor and in general, the larger the margin the less chance of recurrence [1,4]. However, reconstruction becomes more complicated when a greater amount of bone is removed. While all methods of reconstruction have their own unique benefits and drawbacks, the use of an endoprosthesis is associated with earlier weight-bearing and improved functional outcomes [5-8]. The biggest problem with allograft-prosthetic composites is healing at the allogeneic-autologous bone interface [1-6]. In addition, proper fitting of the prosthesis is important for good long-term functional outcomes.

Technological advances have led to the use of computer-assisted surgery and computer-aided design (CAD) in many medical fields including oncology and orthopedics [9-19]. Reports by Wong et al. [10,13] and Khan et al. [11] have indicated that computer-assisted methods can improve the accuracy of resection of malignant bone tumors. Similarly, other authors have reported the utility of CAD for custom endoprosthesis construction [4,18,19].

We have uniquely used preoperative CAD to plan the surgical resection and develop a custom endoprosthesis for patients with malignant bone tumors. The technique allows precise planning of the surgical resection and development of an allograft-prosthetic composite that precisely fits the area of resection. The purpose of this study is to describe the technique and to report the results in 12 patients with malignant bone tumors of the distal femur or proximal tibia.

## Methods

### Patients

This study was been approved by the Institutional Review Board (IRB) of School of Materials Science and Engineering, South China University of Technology, Guangzhou, Guangdong, China. All patients provided written informed consent for the procedures performed.

Patients were recruited between November 2006 and January 2012. Inclusion criteria were distal femur and proximal tibia malignant bone tumor around the knee. Exclusion criteria were: (1) distant metastasis; (2) benign tumor; (3) metastatic tumor around the knee.

This study included 9 males and 3 females with a mean age of 25.3 years (range, 13 to 40 years) with malignant bone tumors. There were 9 cases of tumors in the distal femur and 3 cases of tumors in the proximal tibia. Patient data are summarized in Table 1. In all cases, pathological examination of a tumor specimen was performed before surgery for definite diagnosis. Patients received 2 courses of chemotherapy. One week prior to surgery, computed tomography (CT) and magnetic resonance imaging (MRI) examinations were carried out to obtain two-dimensional (2D) CT and MRI data of the lesion, and preoperative simulation processes including CAD design template assisted tumor resection, allogeneic bone trimming templates, and computer simulation surgical procedure were performed.

**Table 1:** The demographic characteristics of 12 subjects

**Patient**	**Sex**	**Age**	**Diagnosis**	**Chemotherapy**	**Surgery**	**Follow-up (mo)/Status**
**1**	M	19	Left distal femur osteosarcoma	Y	Radical resection, allogeneic bone + full knee reconstruction	74/Alive
**2**	M	39	Right tibia chondrosarcoma	N	Radical resection, allogeneic bone + plate fixation reconstruction	47/Alive
**3**	F	32	Left femur osteosarcoma	Y	Radical resection, allogeneic bone + personalized full knee reconstruction	33/Alive
**4**	M	40	Left femur osteosarcoma and fractures	Y	Radical resection allogeneic bone + personalized full knee reconstruction	Died of lung metastases at 18 months
**5**	F	13	Left tibia osteosarcoma	Y	Radical resection, allogeneic bone + personalized full knee reconstruction	26/Alive
**6**	M	26	Left distal femur chondrosarcoma	N	Radical resection, allogeneic bone + personalized full knee reconstruction	17/Alive
**7**	M	26	Left distal femur osteosarcoma	Y	Radical resection allogeneic bone + personalized knee reconstruction	29/Alive, lung metastases
**8**	M	13	Left distal femur osteosarcoma	Y	Radical resection, allogeneic bone + personalized full knee reconstruction	Died of lung metastases at 1 year
**9**	M	16	Left distal femur osteosarcoma	Y	Radical resection, allogeneic bone + personalized full knee reconstruction	15/Alive
**10**	M	26	Right femur myofibroblastic sarcoma	N	Radical resection, allogeneic bone + femoral nail fixation	12/Alive
**11**	F	31	Giant cell tumor of the left tibia	N	Margin resection, bone cement + plate fixation reconstruction	5/Alive
**12**	M	23	Giant cell tumor of the left tibia	N	Extended resection, allogeneic bone + plate fixation reconstruction	5/Alive

### Computer aided design

Computer simulation of individualized bone tumor resection and reconstruction included three-dimensional (3D) reconstruction of the disease area, identification of tumor resection range, computer-aided design (CAD) surgical template, CAD individualized prosthesis, and computer-simulated bone tumor resection and reconstruction. The 2D CT image data were imported into Mimics 14.0 software (Belgium) to reconstruct a 3D anatomical model of the bone and joint at the site of the lesion. Thin-section 2D MRI image data were imported into Mimics software to reconstruct a 3D model of the region invaded by the tumor, and accurately identify the range of the lesion. Image registration and alignment of the bone and joint anatomical model and the tumor invasion model were performed. Imageware 12.0 (UGS Corporation, USA) was used for the 3D reconstruction of lower limb mechanical parameters, tumor range boundary measurements, design-assisted surgery template, and computer-aided simulation surgery.

The boundary of surgical resection was decided according to the nature of the tumor. Generally, normal bone tissue 3–5 cm distal to the tumor boundary was removed together with the tumor. The distance from the tumor to the articular surface was used to decide whether or not to remove the joint. If the distance was more than 5 cm, the joint was not removed and bone grafting was performed using a large allogeneic bone matching the normal anatomical structure. If the distance was less than 5 cm, the joint was removed and bone grafting using a large allogeneic bone together with an individualized artificial joint was performed.

### Surgical procedure

After induction of general anesthesia, the surgical area was prepared and draped, and the tumor was sufficiently exposed. An auxiliary template was installed to guide accurate tumor resection. The surgical area was soaked by distilled water for 10 min to promote necrosis of free tumor cells due to the low osmolality. Using an allogeneic trimming template, a large allogeneic bone obtained before surgery was trimmed into a 3D shape matching the bone defect after tumor resection. If needed, it was fixed to an individualized metal prosthesis with screws or bone cement to form an individualized prosthesis for repair of the bone defect. The individualized prosthesis for bone defect repair was then implanted into the bone defect area. Bone cement, bone ingrowth, or screw fixation were used to fix the prosthesis to the autogenic bone.

### Postoperative care

In general at 10 days after surgery patients were fitted with a brace and allowed to walk with crutches. Patients were discharged at 14 days after surgery after removal of sutures and there was no evidence of infection. Weight bearing was begun 12 weeks after surgery.

During follow-up, patients were evaluated with International Society of Limb Salvage (ISOLS) scores. The ISOLS system scores 6 categories (pain, overall function, acceptance, supporting tools, walking, and gait). Each category is rated 0–5 with 0 being the worst score and 5 the best (e.g., for the pain category 0 = serious pain and 5 = no pain), for a total score of 30. A score of 24 to 30 is considered excellent, 18 to 23 good, 12 to 17 fair, and < 12 points poor.

## Results

In all cases the tumors were removed successfully, and postoperative pathological examination confirmed clear surgical margins. All 12 patients underwent reconstruction with allogeneic bone and an endoprosthesis modeled using CAD. There were no surgical complications. Postoperative radiographs showed that the range of tumor resection was completely in accordance with the preoperative design, and showed that the morphological reconstruction of the bone defect area was satisfactory with complete bilateral symmetry. The reconstructed structure was very stable with excellent weight-bearing capacity, which resulted in early recovery of physical activity and daily living. The mean follow-up time of the 12 patients was 26.5 months, and as of this report 10 are alive and well. At the last follow-up, the average ISOLS score was 25.8 (range, 18 to 27), and was excellent in 8 cases and good in 4 cases (Table 2).

**Table 2:** International Society of Limb Salvage (ISOLS) scores at final follow-up

**Patient**	**Pain**	**Overall function**	**Acceptance**	**Supporting tools**	**Walking**	**Gait**	**Total score**	**Follow-up (months)**
1	5	3	4	5	5	5	27 (excellent)	74
2	5	3	3	1	3	3	18 (good)	47
3	5	5	5	5	5	5	30 (excellent)	33
4	5	5	5	5	5	5	30 (excellent)	18
5	5	5	4	5	5	5	29 (excellent)	26
6	5	3	4	5	3	3	23 (good)	17
7	5	5	3	5	5	5	28 (excellent)	29
8	5	5	5	5	5	3	28 (excellent)	12
9	5	5	4	5	5	3	27 (excellent)	15
10	4	5	5	1	3	3	21 (good)	12
11	5	5	5	5	5	3	28 (excellent)	5
12	4	5	5	1	3	3	21 (good)	5

### Representative case

#### Computer aided 3D modeling

**Figure 1:**
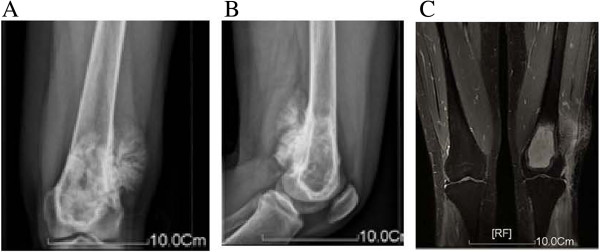
**Data of radiography. A**, **B)** Anteroposterior and lateral radiographs and **C)** magnetic resonance imaging studies were consistent with an osteosarcoma of the left distal femur.

A 31-year-old female was seen for left knee pain and discomfort for 6 months. Radiography suggested an osteosarcoma in the left distal femur, which perforated laterally and posteriorly and formed a mass adjacent to the cortex (Figure 1A, B). MRI and an open biopsy were performed, and the results were consistent with an osteosarcoma adjacent to the cortex in the left distal femur. The lesion was classified as Enneking stage IIB. A three-stage treatment including preoperative chemotherapy, surgery, and postoperative chemotherapy was designed. The surgical strategy was en bloc tumor resection and limb salvage.

**Figure 2:**
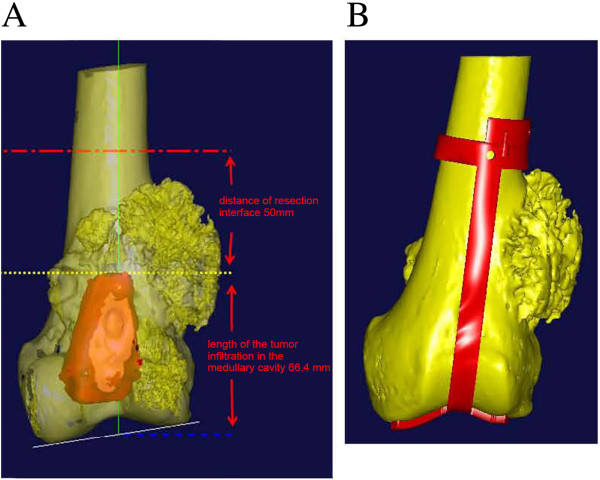
**Measurement and resection of tumor region. A)** The distance between the proximal end of the tumor located in the medullary cavity of the left distal femur and the femoral intercondylar notch was about 66.4 mm. The orange-red area represents the 3D morphology of the tumor region. The distance from the proximal end of the tumor (yellow dotted line) to the planed excision point (orange dotted line) was 50 mm. Thus, a total of 116.4 mm of the distal femur was to be removed. **B)** Auxiliary template to guide tumor resection.

First, bilateral lower-extremity CT and subsequent 3D reconstruction were carried out to establish an anatomical model containing the bilateral femurs, tibias, fibulas, and patellae Second, plain and enhanced MRI was performed to verify the range of tumor invasion, and the 3D morphology of the bone tumor was reconstructed (Figure 1C). Both models were combined and analysis and measurements were carried out after registration and alignment. The distance between the proximal end of the tumor located in the medullary cavity of the left distal femur and the femoral intercondylar notch was about 66.4 mm. Based on oncological principles for en bloc tumor resection, normal bone 5 cm in length in the junction zone was to be removed. Because the length of tumor invasion into the medullary cavity was 66.4 mm, a total of 116.4 mm of the distal femur were to be removed (Figure 2A). A CAD designed auxiliary template was created to guide precise tumor resection during surgery (Figure 2B).

**Figure 3:**
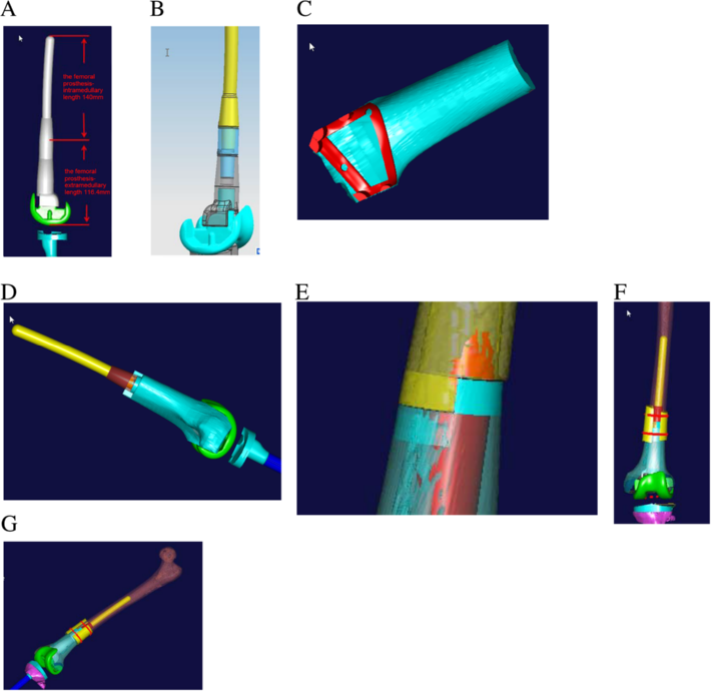
**Design of the femoral prosthesis. A)** Prosthesis parameters. **B)** CAD model of the femoral prosthesis. **C)** Template to trim the allogeneic bone to match the surfaces in the distal end of the femoral prosthesis. **D)** The trimmed allogeneic bone was set in the outer surface of the individualized metal prosthesis to form an individualized femoral prosthesis. **E)** Stepped contact in the junction zone. **F)** Allogeneic bone covering the junction at the distal end of the remaining femur and fixed with a band. **G)** Final 3D model of distal femur reconstruction.

The parameters of the femoral prosthesis were designed according to the morphology of the bone defect and the length of osteotomy. The extramedullary length was 116.4 mm, and the intrameduallary length was 140 mm (Figure 3A). The diameter of the femoral shaft at the site of the osteotomy was 29 mm, the diameter of the medullary cavity at the site of the osteotomy was 17 mm, and the narrowest diameter of the medullary cavity was 15 mm. Based on these parameters, the length of the prosthesis entering the medullary cavity of the residual femur was 146.5 mm. The diameter of the proximal end of the cone-shaped prosthesis was 13 mm, and the diameter of the distal end was 14.5 mm. The extramedullary part of the femoral stem prosthesis was cylinder-shaped. The length was 120.1 mm and the diameter was 20 mm, which was larger than the diameter of the medullary cavity and smaller than the diameter of the femoral shaft at the site of the osteotomy. The CAD designed femoral prosthesis was composed of four parts: femoral condyle (blue), connector (gray), extension rod (blue), fixation rod (yellow) (Figure 3B). A fastening screw was applied between the femoral condyle and the connector. The connector and the extension rod were connected via a taper hole, and an anti-rotation locking plate was also used.

An allogeneic distal femur 15 mm in length was purchased from Auri Biotechnical Company (Taiyun, Shanxi Providence, China). The allogeneic bone was prepared by cobalt 60 irradiation, and was freeze-dried and stored at room temperature until use. The allogeneic femur was cut to an appropriate length using the CAD designed template for trimming the allogeneic femur. Next, the template was used to trim the allogeneic bone such that it matched the surfaces in the distal end of the individualized femoral prosthesis (Figure 3C). The trimmed allogeneic bone was then set in the extramedullary portion of the individualized femoral prosthesis, and bone cement was used for fixation (Figure 3D).

Stepped contact was designed between the allogeneic bone and the residual proximal femur to increase the contact area, reduce stress shielding, and enhance the interface healing (Figure 3E). Moreover, this site was covered with the allogeneic bone, which was fixed by a double wire band (Figure 3F). Conventional prosthetic replacement was carried out in the proximal end of the tibia. A reconstruction model of the distal femur and proximal tibia is shown in Figure 3G.

#### Surgical procedure

**Figure 4:**
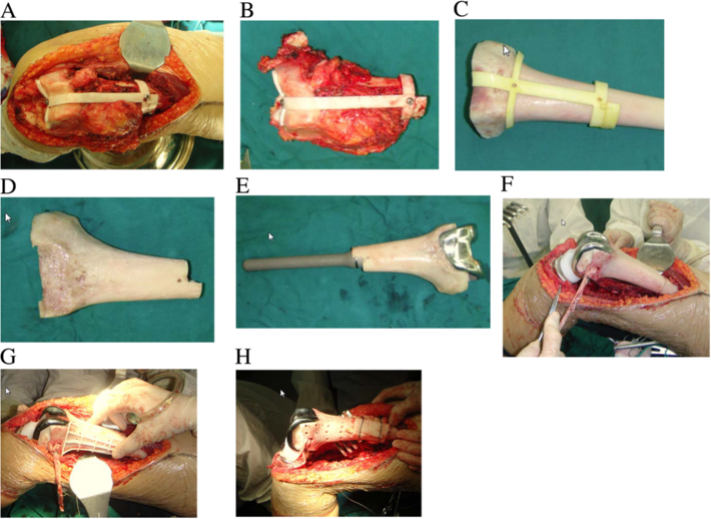
**Intraoperative photos. A)** Auxiliary template for tumor resection was installed. **B)** Resected specimen. **C)** Allogenic bone with template for trimming. **D)** Trimmed allogeneic bone. **E)** The trimmed allogeneic bone with prosthesis installed. **F)** Distal femur implant in place. **G)** Template for drilling holes in the allogenic bone. **H)** Allogeneic bone was used to cover the junction zone and fixed with a double wire band.

The distal femur was exposed, and the auxiliary tumor resection template was installed to guide the osteotomy (Figure 4A) and precise tumor resection (Figure 4B). An allogeneic trimming template was installed on the allogeneic bone (Figure 4C), and the allogeneic bone was trimmed to a 3D shape matching the bone defect area (Figure 4D). An allogeneic tendon was passed through both sides of the distal part of the allogeneic bone for collateral ligament reconstruction. The trimmed allogeneic bone was combined with the prosthesis, and bone cement was applied to fix the related parts to form an individualized prosthesis for bone defect repair (Figure 4E).

**Figure 5:**
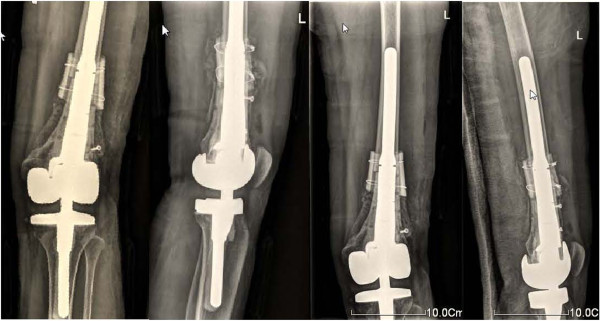
Postoperative radiographs showed good placement of the femoral and tibial prostheses.

Conventional osteotomy was performed 10 mm inferior to the articular surface of the right proximal tibia as determined by 3D modeling. The individualized femoral prosthesis was inserted into the medullary cavity of the femur. Conventional prosthetic replacement was carried out on the tibial plateau to reconstruct the bone and joint structure (Figure 4F). A guiding template for hole-drilling was installed (Figure 4G) to guide drilling on the surface of the allogeneic bone. Allogeneic bone fragments were applied to cover the junction between the patient’s own bone and the allogeneic bone. A double wire was placed for fixation (Figure 4H). Autologous bone fragments were placed in the holes on the surface of the allogeneic bone to enhance bone regeneration. After placement of a drain and wound closure, the knee joint was fixed with a brace. Postoperative X-ray showed that the reconstruction of the bone and joint was extremely accurate, and the prosthesis shape was well-matched (Figure 5).

The total operative time was 5 hours, and the intraoperative blood loss was 600 ml. The amount of postoperative drainage was 600 ml on postoperative day one, and decreased to 50 ml on postoperative day four and the drainage tube was removed. Ankle extension and flexion exercises were begun on postoperative day 1, hip flexion and knee flexion exercises were begun on postoperative day 3, and straight leg raises were begun on postoperative day 5. The patient was encouraged to walk with crutches 10 days after surgery. She was able to walk independently by 3 months after surgery and at the 2-year follow-up she was alive and well without evidence of recurrence.

## Discussion

In this report we have shown the utility of computer-aided analysis, design, and surgical simulation in the management of osteosarcomas in 12 patients. The techniques allowed precise resection of the lesion and the sacrifice of a minimal amount of normal bone and the construction and placement of an accurate fitting endoprosthesis. One week after surgery, computed tomography and magnetic resonance imaging (MRI) examinations were applied to patients’ lower extremity to obtain two-dimensional (2D) CT and MRI data. Postoperative radiographs confirmed limb length, resection boundaries, and other anatomical parameters consistent with the preoperative planning. Additionally, re-applied with CAD design, three-dimensional reconstructions of lower limb joints, allogeneic bone implants and total knee prosthesis were established. To compare the post-operative CAD design with the preoperative CAD design, the whole appearances, the length of the limb, the line of force of limbs are consistent with preoperative planning. In all cases, complete resection of the lesion with clear surgical margins was achieved. The technique, however, requires specialized equipment and expertise.

Surgical resection of the lesion is a critical part in the treatment of malignant bone tumors. The purpose of surgery is to remove the tumor sufficiently to reduce the rate of local recurrence and distant metastasis [1-4]. Although a greater range of excision will decrease the likelihood of recurrence, extensive resection may increase the difficulty of bone and joint reconstruction. Selection of an appropriate range of surgical resection or an accurate surgical boundary can remove the tumor lesion completely and maximally preserve the normal bone structure.

In the past, tumors were removed based on visual identification of the margins, ruler measurement, or palpation, and the actual resection range varied from the preoperative design. Surgical navigation systems were introduced to resolve this problem; however, they are complicated, time-consuming, and associated with a high initial cost [20]. The use of computer-assisted imaging can accurately provide a 3D image of the extent of a tumor to provide for complete resection while salvaging the maximal amount of normal bone [10,11]. Wong et al. [13] used computer-aided bone resection in 20 patients with 21 malignant bone tumors and clear surgical margins were achieved in all cases and the achieved bone resection was within 2 mm of the planned resection in all cases. In another study of 8 patients, Wong et al. [21] reported that computer-assisted surgery was useful for the planning and execution of joint-preserving tumor resection. We applied CAD to create an auxiliary tumor resection template to guide intraoperative tumor resection and achieved excellent outcomes. Postoperative evaluation proved that the template-guided tumor resection is very precise.

Prior studies have reported the production of a solid model with rapid prototyping technology based on CT scans, and then use of the model for prosthesis design and simulated surgery [10,15,16,19]. In this study, we also used CAD to prepare an allogenic bone graft and accurately design a custom endoprosthesis. A 3D model of the allogenic bone was prepared from CT scan data, and bone defect reconstruction and the outcome of bone defect repair were simulated before surgery. The allogeneic bone was trimmed to a 3D shape matching the bone defect according to the CAD auxiliary trimming template. The trimmed allogeneic bone and the individualized metal prosthesis were combined to form a custom prosthesis, which had the strength of a metal prosthesis for early weight-bearing. The 3D modeling allowed the allogenic bone to fit the bone defect area precisely. This not only enhances the early recovery of local mechanical support, but also accelerates the healing speed of the interface and reduces operative time. By designing an individualized prosthesis it was assured that it would match the residual bone, the mechanical strength of the integrated prosthesis was known, and by simulating the installation before surgery any potential problems could be identified, thus reducing operative time and complications.

There are a number of theoretical advantages to the use of computer simulation of bone tumor resection and subsequent reconstruction. 1) By simulating the surgery, potential problems can be identified and remedial methods and preventive measures can be considered. 2) Advantages and disadvantages of various methods can be compared to determine the best one and constantly improve the surgical procedure. 3) The surgical team can exchange opinions with respect to the surgical procedure and all members can become familiar with the technique. 4) The simulation can be shown to the patient and family members to provide them a better understanding of the surgery and reduce their anxiety. We utilized a completely digital simulation such that the surgical procedure and the prosthesis design could be freely adjusted during simulation to decide the best treatment method and produce an individualized prosthesis according to the design.

There are few reports of the combined use of an endoprosthesis and allogeneic bone [22,23]. In the current study, the prosthesis surface was covered with allogeneic bone to increase the strength of the bone and joint structure, and improved the attachment of peripheral soft tissue to convert the previous hinged total knee arthroplasty into a procedure close to a conventional prosthetic replacement. The semiconstrained knee design with collateral ligament reconstruction that was used, rather than a rotating hinge knee design that is common in bone tumor reconstruction around the knee, provides a large segment of allogeneic bone around the prosthesis, and ligament reconstruction at the same time provides lateral stability of the prosthesis. Allogeneic bone combined with a non-hinged prosthesis provides reduced stress and less occurrence of loosening and also allows earlier physical activity and better recovery of knee function. The use of a CAD template for the trimming allogeneic bone to exactly match the bone defect allowed for improved contact between the autologous bone and the allogeneic bone to enhance bone union.

There are limitations of this report that should be considered. The primary limitations of this study are the small number of cases and the relatively short follow-up time. In addition, no control group or comparison group was included.

## Conclusions

The results of this study indicate the utility of computer-aided design and modeling for the surgical management of bone tumors and subsequent limb reconstruction. The method provides accurate tumor removal with the salvage of a maximal amount of unaffected bone and precise endoprosthesis reconstruction. Though still in their infancy, computer-aided management of bone tumors holds great promise for providing good outcomes and functional recovery.

## Abbreviations

CAD: Computer-aided design; CT: Computed tomography; MRI: Magnetic resonance imaging; 2D: Two-dimensional; 3D: Three-dimensional.

## Competing interests

The authors declare that they have no competing interests.

## Authors’ contributions

HWD: guarantor of integrity of the entire study; study concepts; study design; manuscript preparation; manuscript editing; manuscript review. GWY: data acquisition; data analysis; manuscript editing; manuscript review. QT: definition of intellectual content; clinical studies; manuscript preparation. BL: literature research; data acquisition; data analysis; statistical analysis; manuscript editing. JJS: clinical studies; manuscript preparation. HW: statistical analysis. YJW: experimental studies. All authors read and approved the final manuscript.

## Pre-publication history

The pre-publication history for this paper can be accessed here:

http://www.biomedcentral.com/1471-2474/14/331/prepub
